# The Diverse Roles of the Global Transcriptional Regulator PhoP in the Lifecycle of *Yersinia pestis*

**DOI:** 10.3390/pathogens9121039

**Published:** 2020-12-11

**Authors:** Hana S. Fukuto, Gloria I. Viboud, Viveka Vadyvaloo

**Affiliations:** 1 Clinical Laboratory Sciences Department, School of Health Technology and Management, Stony Brook University, Stony Brook, NY 11794, USA; gloria.viboud@stonybrook.edu; 2 Paul G. Allen School for Global Animal Health, Washington State University, Pullman, WA 99164, USA; vvadyvaloo@wsu.edu

**Keywords:** PhoP, *Yersinia pestis*, transcription, insect vectors, flea, phagocytes, plague

## Abstract

*Yersinia pestis*, the causative agent of plague, has a complex infectious cycle that alternates between mammalian hosts (rodents and humans) and insect vectors (fleas). Consequently, it must adapt to a wide range of host environments to achieve successful propagation. *Y. pestis* PhoP is a response regulator of the PhoP/PhoQ two-component signal transduction system that plays a critical role in the pathogen’s adaptation to hostile conditions. PhoP is activated in response to various host-associated stress signals detected by the sensor kinase PhoQ and mediates changes in global gene expression profiles that lead to cellular responses. *Y. pestis* PhoP is required for resistance to antimicrobial peptides, as well as growth under low Mg^2+^ and other stress conditions, and controls a number of metabolic pathways, including an alternate carbon catabolism. Loss of *phoP* function in *Y. pestis* causes severe defects in survival inside mammalian macrophages and neutrophils in vitro, and a mild attenuation in murine plague models in vivo, suggesting its role in pathogenesis. A *Y. pestis* *phoP* mutant also exhibits reduced ability to form biofilm and to block fleas in vivo, indicating that the gene is also important for establishing a transmissible infection in this vector. Additionally, *phoP* promotes the survival of *Y. pestis* inside the soil-dwelling amoeba *Acanthamoeba castellanii*, a potential reservoir while the pathogen is quiescent. In this review, we summarize our current knowledge on the mechanisms of PhoP-mediated gene regulation in *Y. pestis* and examine the significance of the roles played by the PhoP regulon at each stage of the *Y. pestis* life cycle.

## 1. Introduction

*Yersinia pestis*, a gram-negative bacterium that causes plague, emerged as a subclone of *Yersinia pseudotuberculosis* as recently as 5000–6000 years ago [1,2,3,4]. Despite the genetic similarity [5], *Y. pestis* has evolved a distinct and more complex life cycle compared to its ancestor. While *Y. pseudotuberculosis* normally persists in the soil and is transmitted through contaminated food and water, *Y. pestis* primarily infects rodents and is transmitted to other mammals by fleas [6,7], and has poor survivability in the soil by itself [8,9,10]. When the flea vector takes a blood meal from an infected animal, the bacteria enters the digestive system and forms a biofilm that blocks the flea foregut. As the blocked flea tries to feed on a new mammalian host, it transmits the bacteria by regurgitating into the bite site [6]. *Y. pestis* occasionally infects humans through flea bites, causing bubonic plague. The bacteria can also be transmitted through an aerosol route to cause more life-threatening pneumonic plague [7]. If untreated, both bubonic and primary pneumonic plague can lead to septicemic plague and death. While *Y. pestis* causes high mortality in mammalian hosts during the epizootic phase, it can also persist for a long time without causing fatal outbreaks during the inter-epizootic/quiescent phase [10]. It is commonly thought that the bacteria survive by circulating between resistant populations of rodents and fleas. However, it has also been suggested that the bacteria may survive by residing inside free-living amoeba in the soil [10]. Thus, *Y. pestis* encounters a wide range of conditions during its life cycle and needs to respond rapidly to the changes in order to achieve successful maintenance and propagation.

Bacterial two-component systems are transcriptional regulators that play critical roles in the adaptation of the bacteria to changing habitats. They can sense various environmental cues and orchestrate complex regulatory networks to facilitate transcriptional responses. The analysis of the *Y. pestis* CO92 genome has revealed up to 29 predicted two-component systems, including four pseudogenes [11,12]. Of these, the PhoP/PhoQ (PhoPQ) two-component system has been most extensively studied as a potential virulence determinant. Studies have shown that the function of PhoP, the response regulator in the PhoPQ system, is important for various stages of *Y. pestis* life cycle, including survival inside the mammalian phagocytes, biofilm formation inside the flea gut, and survival inside the soil-dwelling amoeba. In this review, we summarize our current knowledge on the signals that activate *Y. pestis* PhoP in different host environments, the genes and molecular pathways that are regulated by PhoP, and different ways by which the PhoP regulon contributes to the adaptation of *Y. pestis* to various stages of its life cycle.

## 2. *Y. pestis* PhoP Regulatory Networks

PhoPQ is a two-component system that plays a crucial role in virulence and the adaptation to harsh conditions in many Gram-negative bacteria. PhoPQ consists of the sensor histidine kinase/phosphatase PhoQ and the response regulator PhoP. PhoQ detects changes in the periplasmic environment and transduces a signal that promotes kinase activity in its cytoplasmic domain. The phosphoryl group is then transferred to activate PhoP in the cytoplasm. Phosphorylated PhoP binds to sequences containing two direct (T/G)GTTTA repeats separated by five nucleotides in the promoter region of a number of genes, activating or repressing their transcription [13]. 

**Figure 1:**
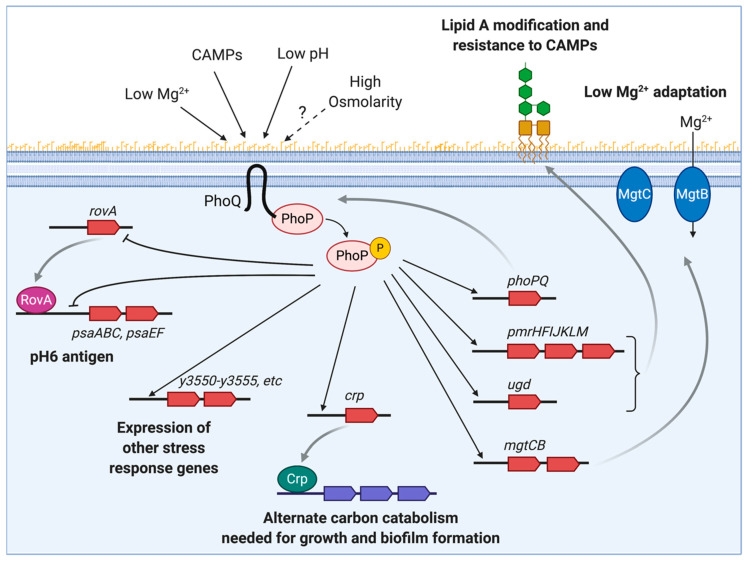
The *Yersinia pestis* PhoPQ regulatory network. The sensor kinase PhoQ detects changes in the periplasmic environment, such as low Mg^2+^, cationic antimicrobial peptides (CAMPs), low pH and possibly high osmolarity, and phosphorylates the response regulator PhoP in the cytoplasm. Phosphorylated PhoP binds to the promoter region of a number of genes, activating or repressing their transcription. PhoP-regulated genes include *pmrHFIJKLM/arnBCADTEF* and *ugd*/*pmrE*, which are necessary for the modification of the lipid A moiety of *Y. pestis* lipooligosaccharide and resistance to CAMPs; *mgtBC* genes that are involved in adaptation to low Mg^2+^ environment; a global regulator *crp* that coordinates genes necessary for carbon uptake and catabolism, as well as biofilm formation; and other stress response genes, such as the *y3550–y3555* operon (locus tags based on KIM strain). PhoP also regulates its own expression and represses pH6 antigen-encoding *psaABC* and *psaEF* genes either directly or indirectly by repressing RovA. This figure was created with BioRender.com.

During infection, the PhoPQ system can sense different environmental stresses, such as low magnesium (Mg^2+^), the presence of cationic antimicrobial peptides (CAMPs) and low pH (Figure 1). Positively charged Mg^2+^, CAMPs, and H^+^ interact with highly acidic residues in PhoQ’s periplasmic sensor domain. Under physiological concentrations, Mg^2+^ is bound to inactive PhoQ, and in Mg^2+^ limiting conditions, the cations dissociate and PhoQ gets activated [13]. Similarly, CAMPs activate the system by displacing Mg^2+^ from PhoQ metal binding sites [14]. Hydrogen ions also bind to the PhoQ sensor domain, but do not directly compete for binding to divalent cation-binding sites [15]. Additional stimuli associated with the host, such as hyperosmotic stress and disruption of the oxidizing environment of the periplasm, have been proposed to stimulate PhoQ in *E. coli* [16,17].

PhoP regulates the activity of a number of genes and operons that govern a wide variety of cell processes in *Y. pestis*. Transcriptome analysis revealed that 0.5–2.0% of the whole *Y. pestis* genome is either activated or repressed by PhoP [18,19]. However, PhoP only binds to the promoters of a limited number of genes or operons, suggesting that most PhoP targets are indirectly regulated [20]. The direct targets of PhoP include those involved in adaptation to a low-Mg^2+^ environment (*mgtCB*), protection against reactive oxygen species (*sodA*, *sodB*, *sodC*, *katA*, and *ahpC*) and other stresses, and resistance to antimicrobial peptides (*arnBCADTEF/pmrHFIJKLM*, *ugd/pmrE*, and *pagP*) [20] (Figure 1). Antimicrobial peptides are critical components of the host innate immune response that bind to and destabilize the outer membrane of Gram-negative bacteria. To decrease the binding of the positively-charged CAMPs, *Y. pestis* uses the gene products of the PhoP-regulated *pmrHFIJKLM* (also termed *arnBCADTEF* or *pbgP* operon) and *ugd* (also termed *pmrE*) genes to add a 4-aminoarabinose to the lipid A, thereby neutralizing the negative charge of the outer membrane [21,22]. Another *phoP*-regulated gene, *pagP*, encodes a lipopolysaccharide (LPS) modifying enzyme that mediates the transfer of a palmitate group to the lipid A in *Y. pseudotuberculosis* and *Yersinia enterocolitica*. This modification increases the Toll-like receptor 4(TLR4)/myeloid differentiation factor 2 (MD-2)-mediated innate immune recognition of these pathogens during infection. However, *Y. pestis* lacks the palmitoylated lipid A species [23]. A recent study shows that *Y. pestis* has a single nucleotide polymorphism (SNP) that renders the enzyme inactive [24]. It is proposed that the loss of a functional PagP during *Y. pestis* evolution resulted in increased survival of the bacteria in the mammalian hosts by evasion of the innate immune response.

*Y. pestis* PhoP governs various cellular pathways indirectly by acting on a set of regulators [20,25]. Beside regulating its own expression, PhoP activates the transcription of the global regulator, cyclic AMP receptor protein (*crp*), which controls sugar uptake and catabolism, and modulates a wide variety of *Y. pestis* genes, including those involved in quorum sensing, iron acquisition, biofilm formation, and the expression of several virulence factors [25,26]. PhoP also controls the expression of the global virulence regulator RovA, which activates expression of the antiphagocytic pH6 antigen in *Y. pestis*, and the adhesin invasin in *Y. pseudotuberculosis* and *Y. enterocolitica* [27,28]. While *rovA* expression is self-activated, it is repressed by PhoP binding to its promoter region [27]. 

The evolution of gene regulation in bacteria results in major differences between related organisms. Although *Y. pestis* and *Salmonella* PhoP are highly homologous, their individual regulons are considerably different. One example is the highly conserved type III secretion system (T3SS), which is essential for virulence in the two pathogens. The T3SSs encoded in the *Salmonella* pathogenicity island (SPI)-1 and SPI-2 are highly regulated by PhoP. In *Salmonella*, induction of endocytosis by epithelial cells governed by SPI-1 is repressed by PhoP’s negative effect on the regulator HilA, and SPI-2-mediated intramacrophage survival is activated by the action of PhoP on SsrB, a regulator that binds to the promoters of all SPI-2 functional gene clusters [29,30]. Notably, activation of the T3SS in *Yersinia* does not appear to be regulated by PhoP. 

While PhoP proteins from *Yersinia* and *Salmonella* both regulate a common set of core ancestral genes, they differentially transcribe species-specific genes in the two pathogens [31]. Similarly, both conserved and unique PhoP binding sites are found in *Y. pestis* and *Salmonella.* The conserved promoters, called prototypical class II, regulate *Salmonella* ancestral genes, such as Mg^2+^ transport gene *mgtA*, but not genes acquired horizontally. On the other hand, *Salmonella’s* horizontally acquired *ugtL* gene, which is required for low pH resistance, has a PhoP box with an inverted orientation that is not present in *Yersinia* [31]. Most PhoP boxes in *Yersinia* resemble the organization of the class II promoter with respect to its orientation and its distance from the -10 region. Other *Yersinia* PhoP DNA binding sites, such as that of *mgtC*, are located further upstream of the transcription initiation site. 

In addition to different promoter structures, *Yersinia* and *Salmonella* use different PhoP regulatory pathways to control the genes encoding conserved proteins. The arabinose modification of Lipid A in *Salmonella* is regulated by the PhoP-activated PmrD protein. PmrD post-translationally activates PmrA, which binds to the promoter of the 4-aminoarabinose biosynthetic genes. Because *Yersinia* lack PmrD, regulation of the *pmr* operon and *ugd* in a low-Mg^2+^ environment occurs by direct binding of PhoP to a second promoter in those genes [21]. It reflects how evolutionary changes in the PhoP regulon allows the pathogen to transcribe newly acquired genes and maintain control of ancestral ones. 

## 3. Role of *phoP* in Intracellular Replication in Mammalian Hosts

*Y. pestis* is a facultative intracellular pathogen. During *Y. pestis* infection, bacteria are mainly found extracellularly within the tissues of an infected host. To inhibit phagocytosis and suppress proinflammatory cytokine production by host immune cells, *Y. pestis* expresses an array of virulence factors, including T3SS and Yop effector proteins encoded on a virulence plasmid (pCD1), as well as a capsule composed of F1 protein [32,33]. However, the expression of these factors is suppressed when *Y. pestis* is inside the flea [34], so at the initial stage of infection, the bacteria are more readily taken into the professional phagocytes. Therefore, it has been proposed that the host phagocytes may provide a replicative niche and a vehicle for dissemination of *Y. pestis* before the pathogen can replicate more robustly in the extracellular environment [35]. Consistent with this hypothesis, *Y. pestis* is found inside host macrophages in experimentally infected animals [36,37,38,39,40]. More recently, in vivo tracking studies using intravital confocal microscopy and flow cytometry in murine intradermal models of bubonic plague have shown that neutrophils, and later resident macrophages and dendritic cells (DCs), to a lesser extent, are recruited to the initial site of infection within several hours of the pathogen inoculation [41,42,43]. In these studies, the bacteria are often found to be associated with or inside macrophages and neutrophils [41,42].

Various in vitro studies have also demonstrated that *Y. pestis* can survive and replicate within macrophage phagosomes [19,39,44,45], and that *phoP* is essential for this ability. The *phoP* deletion mutants of *Y. pestis* exhibit a severe defect in growth inside murine macrophages, as well as growth under in vitro conditions that mimic the intracellular environment: low pH, oxidative stress, low Mg^2+^, high osmolarity, and the presence of antimicrobial peptides [19,44,46]. Transcriptome analysis has shown that many PhoP-regulated genes, including the members of the *pmr* operon, *ugd*, and *mgtC*, are more highly expressed in *Y. pestis*, replicating inside macrophages compared to the bacteria replicating in the tissue culture media [47]. Inactivation of these PhoP-regulated genes reduces the ability of the bacteria to replicate inside murine macrophages [19,48], confirming that they are critical for the adaptation of *Y. pestis* to the intracellular environment. Surprisingly, deletion of *mgtB*, the PhoP-regulated gene encoding for an Mg^2+^ transporter, does not affect intracellular replication of *Y. pestis*, although the mutant is attenuated in murine infection models, suggesting that MgtB may have an additional role in *Y. pestis* pathogenesis outside of intracellular survival [49]. Inside macrophages, *Y. pestis* resides in spacious phagosomes, some of which have acquired double membranes and the characteristics of autophagosome [50]. Like other intracellular pathogens that survive inside macrophages by inhibiting phagosomal maturation, *Y. pestis* blocks acidification and remodels its phagosome by recruiting components of the host endocytic pathway Rab1b, Rab4, and Rab11 [50,51,52]. However, there is no evidence that *phoP* plays a part in this process [50]. 

*Y. pestis* is also able to survive inside neutrophils, the first immune cells to be recruited to the site of infection. Though less permissive than macrophages, about 10–15% of the bacteria internalized by human neutrophils survive and replicate intracellularly [22]. Deletion of *phoP* leads to defective survival inside human neutrophils and increased sensitivity to the components of α-granules produced by these cells, including several CAMPS in vitro [22]. *Y. pestis*-containing neutrophils can be internalized into macrophages in a process called efferocytosis, thereby suppressing the secretion of inflammatory cytokines while promoting the expression of anti-inflammatory cytokine 1L-1RA [53]. Therefore, it has been proposed that neutrophils may provide a path for *Y. pestis* to infect macrophages while minimizing inflammatory responses [53].

Despite the well-established role of *phoP* in the intracellular replication of *Y. pestis*, the extent of its contribution to the overall virulence in mammalian hosts is less clear. In one study, the *Y. pestis* strain GB Δ*phoP* showed a 75-fold increase in the LD50 compared to the wild type in a murine bubonic plague model via subcutaneous infection [44]. The strain KIM5+ Δ*phoP* was also attenuated in a murine pneumonic plague (aerosol) model (Bliska, J. B., unpublished results [54]), suggesting that *phoP* is important for the virulence of *Y. pestis.* However, in another study, there was no difference in the LD50 between a CO92 wild-type strain and the corresponding Δ*phoP* mutant in murine bubonic (subcutaneous inoculation) and pneumonic (aerosol) plague models [55]. Only an increase in time to death was observed in the bubonic plague model at lower dosages, indicating that the role of *phoP* in *Y. pestis* virulence may be minor. One contributing factor for the difference in observed phenotypes could be that these studies used different strains of *Y. pestis* (GB vs. CO92) and mice (BALB/c vs. Swiss Webster mice) [44,55]. Moreover, these studies used subcutaneous injection, while the natural fleabites target the intradermal layer of skin. To mimic flea-mediated infection more closely, murine intradermal infection models have been established in the last several years [41,43]. It would be interesting to know whether testing *phoP* mutants using the latter model would reveal a role of this gene in bacterial dissemination from the initial site of infection that was not obvious in the earlier studies of this mutant.

The relatively mild attenuation of *Y. pestis phoP* mutants is similar to the effect of *phoP* deletion on virulence of *Y. pseudotuberculosis* (40–100× increase in LD50) [55,56], but is in contrast to the severe attenuation of *phoP* mutants in *Salmonella* [57,58,59]. This is probably because *Salmonella* PhoP controls a wider range of critical virulence genes than *Y. pestis* PhoP, including the SPI-1 and SPI-2 genes [29,30]. In addition, the requirement for the intracellular phase of *Y. pestis* in its pathogenesis is controversial. Although neutrophils are recruited to the site of *Y. pestis* injection and the bacteria are found nearby or inside these cells, neutrophil depletion did not alter dissemination or the virulence of *Y. pestis* in murine intradermal [41,43] and intranasal [60] infection models using fully virulent strains. Tracking studies have shown that *Y. pestis* reaches draining lymph nodes as quickly as 10 min after intradermal inoculation, without using macrophages and neutrophils as carriers [43], suggesting that these cells are not required for trafficking or the pathogenesis of *Y. pestis*. However, in another murine subcutaneous infection model, using an attenuated strain of *Y. pestis* KIM5-, lacking the pigmentation (*pgm*) locus necessary for infection via peripheral routes, massive infiltrates of DCs and macrophages were observed at the site of injection, many harboring intracellular bacteria [61]. In this study, the migration of *Y. pestis*-containing phagocytes via lymphatic vasculature was found to be critical for bacterial dissemination and virulence [61]. In yet another murine intravenous infection study, depletion of macrophages and DCs led to the reduced colonization of spleen and liver by *Y. pestis* [62]. These results support the idea that macrophages and DCs are important for the early replication and dissemination of *Y. pestis*. Further investigation is necessary to determine the exact role of the intracellular stage in *Y. pestis* pathogenesis, as well as the contribution of *phoP* to the overall virulence of *Y. pestis* in mammalian hosts. Also, of interest is whether *phoP* plays any additional role outside of intracellular replication during infection of mammalian hosts.

## 4. Biofilm and Flea Colonization

Unlike its seemingly uncertain contribution to development of infection in the mammalian host, PhoP appears to be important for coordinating the flea-associated stage of *Y. pestis* to enable transmission [34,63,64]. Inside fleas, *Y. pestis* forms an adherent biofilm in the foregut to prevent bloodmeal passage to the midgut, and this blockage enhances *Y. pestis* transmission by inducing the flea’s feeding attempts and the subsequent regurgitation of the bacteria from the biofilm [6]. PhoP function is required for the formation of sufficiently cohesive biofilm, which robustly attaches to the flea foregut to maintain normal blockage. A *phoP* mutant instead forms aggregates of fragile biofilm localized to the midgut, and is therefore less likely to be transmitted [63]. Although PhoP does not directly regulate genes that control biofilm production, it directly activates the expression of the carbon catabolite regulator *crp* [25], which coordinates utilization of secondary carbon nutrient sources to support a marked growth spurt and strong biofilm formation [25,26,65]. Genes involved in the uptake and catabolism of secondary sugars like galactose, as well as the pentose sugars, ribose, arabinose, and xylose, are expressed in fleas blocked with the designated wild-type, *Y. pestis* KIM (pCD1- *pgm+*) strain [34]. In addition, genes encoding ribose ABC transporter proteins (*y3243/rbsA*, *y3345*, and *y3346* in KIM strain) are down-regulated in the *phoP* mutant infecting fleas [64]. These results are consistent with a role of PhoP in mediating Crp-dependent regulation of alternate sugar use during flea infection.

Mildly acidic pH (~6.6) and hyperosmolarity (~500 mOsm) of the flea gut milieu are considered to be the stress signals encountered by *Y. pestis* during insect infection [66,67]. PhoP expression is closely linked to management of these stresses in the flea gut, as demonstrated in comparative transcriptomic studies between a *phoP* mutant and wild-type strain during flea infection [64]. A number of stress-response genes are differentially regulated in the *phoP* mutant, including a polycistronic operon comprised of six low-GC-content genes (*y*3555/*aspB*-3554/*nhaC*-*y3553*-*y3552*-*y3551*/*ridA*-*y3550* in KIM). [64]. These genes have putative roles in aromatic amino acid metabolism and are stimulated by hyperosmotic salinity [67]. Together with *phoP* and *y3555*, *y3553* and *y3550* rank among the mostly highly expressed genes in wild-type *Y. pestis* during flea blockage [34]. Another example is that of the YhcN family genes (*y0666* and *y1667* in KIM), which normally display induced expression and promote bacterial aggregation at low pH conditions in vitro [64]. These genes are highly expressed in fleas blocked from a wild-type infection [34], and in a *phoP* mutant their expression is further induced, presumably to manage the acidity in the flea gut in the absence of PhoP. PhoP binding motifs have been identified by bioinformatic analysis in the promoter regions of *y0666* and *y3555/aspB* [68], predicting that these genes are directly regulated by PhoP. 

Known PhoP-repressed genes *psaABC* and *psaEF*, which are involved in production of the pH 6 antigen [28,69], also have increased expression in the *phoP* mutant during flea infection, suggesting that they are derepressed by the absence of PhoP. In addition to direct repression, PhoP indirectly represses *psa* gene expression by downregulating the transcription of *rovA*, whose gene product positively regulates *psa* gene expression [27,28]. However, while wild-type bacteria in the blocked fleas show significant down-regulation of *rovA* [27,28] only a small increase in *rovA* expression occurs in the *phoP* mutant during flea infection. The expression of *psa* genes is also stimulated by acidic conditions in vitro [27,28]. Thus, the activation of *psa* transcription in the *phoP* mutant is likely a combined effect of direct derepression by PhoP, induction through de-repression of the activator RovA, and low pH. It is proposed that the fimbrial structure that constitutes the pH 6 antigen may interfere with the cohesiveness of the *Y. pestis phoP* mutant flea biofilm [64]. Whether this is the reason that the pH 6 antigen is generally repressed in wild-type blocked fleas by significant down-regulation of *rovA* and high expression of *phoP* [34] is unknown. 

Other conserved PhoP-regulated genes, *pmrHFIJKLM* and *ugd*, are downregulated in the *phoP* mutant colonizing fleas. However, their roles in PhoP-mediated flea infection are elusive. This is because the deletion of *ugd* and *pmrA*, a response regulator that is thought to control the *pmr* operon, along with PhoP in *Y. pestis*, does not affect flea infection dynamics during single infection [63], but mutants defective in lipid A modification appear to be required for early flea infection when competing with a wild-type strain [70]. It is unknown whether *Y. pestis* elicits a response to flea antibacterial CAMPs. Further to this is that decreased expression of *mgtC* observed during *phoP* mutant flea infection appears to be innocuous, as the flea Mg^2+^ concentration range is not considered to be low [63].

Multiple stress adaptation genes, not confirmed to be directly regulated by PhoP, are induced in the *phoP* mutant in fleas [64]. This suggests that a compensatory stress response is activated in order to counter unresolved stress due to the loss of PhoP. General stress response genes involved in osmotic stress (*kdpA*, *kdpB*) and heat shock response (*ipbA*, *ipbB*, *rpoH*, *htpX*, and *dnaK)* are examples of such induced genes. Additionally, the expression of L-glutamate group amino acid utilization genes (e.g., *glnHPQ*, *gltJKL*, *astBD*, *argD*, and *hutHU*) is significantly decreased in *phoP* mutant infecting fleas. These are metabolic genes that are implicated in the alleviation of osmotic stress and are predominantly expressed in wild-type blocked fleas [34]. A plethora of other metabolic genes that are normally induced in these blocked fleas are also down-regulated during *phoP* mutant flea infection. Interestingly, a few toxin–antitoxin module encoding genes (*mqsR*, *mqsA*, *higB2*, *y1074*, and *y1075)* are generally found to be induced under stress conditions like glucose and amino acid starvation, hyperosmolarity, and antibiotic exposure also show notably increased expression in the *phoP* mutant in fleas [71]. Expression of stress-related toxin–antitoxin modules is often associated with the formation of persister cells. [72,73]. Persister cells usually comprise 1% of biofilms and are non-growing, metabolically inactive cells that are recalcitrant to environmentally stressful conditions. This supports the notion that the reduced expression of metabolic genes may be a consequence of the *phoP* mutant bacteria attempting to maintain viability in a harsh environment by minimizing metabolic processes. Accordingly, a *phoP* mutant exhibits a severe defect in competitive fitness compared to the wild type in a flea co-infection assay, indicating that this mutant is physiologically impaired [74,75]. Together, in fleas, PhoP plays a critical role in coordinating physiological adaptation to low pH and osmotic stress, in a manner that preserves active metabolic processes that support vigorous biofilm-mediated flea blockage. 

Much of the current PhoP transcriptional response in fleas represents a snapshot at two weeks post-*Y. pestis* infection, when bacteria are presumably not actively replicating. Immediately after fleas acquire a *Y. pestis* infection from feeding on infected blood, the bacteria begin replicating throughout the period of a week to 10 days [76,77]. Fleas start to block 3–5 days post-infection, with the numbers of blocked fleas peaking around 12 days post-infection [78]. Afterwards, fewer fleas are blocked, in accordance with the absence of bacterial replication [76]. One surprising observation in the comparative transcriptomic studies between the *phoP* mutant and wild-type infected fleas is that the expression of the PhoP target, *crp*, is not significantly altered. Given that Crp dictates optimal growth and production of bacterial biofilm, this is unexpected. However, Crp may then only function in the initial two weeks post-infection, in synchrony with active bacterial growth and biofilm blockage development. As such, investigations into the role of PhoP during the first week of active bacterial replication in fleas may provide more comprehensive insight into its role in *Y. pestis* flea infection and biofilm blockage. 

## 5. Amoeba as a Potential Host While the Pathogen Is Quiescent

While the infectious cycle of *Y. pestis* is well-studied, it is not known where and how this pathogen persists naturally during inter-epizootic periods. It has been hypothesized that amoebae serve as reservoir hosts for *Y. pestis* during this quiescent phase [10]. Recent experimental studies show that *Y. pestis* can survive for up to a week within spacious phagosomal compartments in the free-living, soil-dwelling amoeba *Acanthamoeba castellani* [79]. In other bacterial pathogen–amoeba interactions, the composition of the intracellular niche and the factors required for intracellular survival of bacteria are often conserved in both mammalian phagocytes and their phagocytic amoeba counterparts. This is no different for *Y. pestis*, as PhoP is required for intracellular survival in *A. castellani*, with no viable *phoP* mutant cells recovered beyond 24 h post-invasion of amoeba [79]. However, because the *Yersinia*-containing amoebal phagosome does not appear to fuse with the lysosome, it remains unknown if this vacuole has sufficiently low pH to require a PhoP-mediated response. It is also unknown if PhoP-triggering conditions, such as low Mg^2+^ and CAMP expression, are present in the *Y. pestis*-containing amoeba. The mechanism by which *Y. pestis* interacts with amoebae and exploits this potential reservoir niche remains a fascinating question worthy of further exploration. 

## 6. The Effects of a *phoP* SNP on the PhoP Function and the Evolution of *Y. pestis* Virulence

During its emergence as a highly virulent, flea-borne pathogen, *Y. pestis* is thought to have acquired unique characteristics critical for the infection of mammalian hosts and flea vectors through gains and losses of numerous genes, genomic rearrangements, and the incorporation of two plasmids (pMT1 and pPCP1) [5,80]. Additionally, the comparative analysis of both the extant and the ancient *Y. pestis* genomes recovered from archaeological sites suggests that small sequence changes (such as SNPs) in key genes also contributed to the increased virulence and transmissibility of *Y. pestis*. Consistent with this idea, it has been shown that a single amino acid change in the plasminogen activator protease Pla led to the increased ability of *Y. pestis* to disseminate within its mammalian hosts and cause bubonic plague [81], while inactivating mutations in the three genes controlling biofilm formation enhanced the transmissibility of *Y. pestis* by fleas [82]. 

Interestingly, the *phoP* gene also acquired a SNP during *Y. pestis* evolution [83]. *Y. pestis* strains are classified into several phylogenetic branches based on their genome sequences. Branch 0 is rooted in *Y. pseudotuberculosis*, and includes the ancestral/basal strains such as Angola and Microtus. Branches 1 through 4 diverged from branch 0, and include strains associated with the major pandemics of the past, as well as KIM and CO92 [84]. The SNP in *phoP* was acquired near the polytomy where branches 1-4 diverged from branch 0, and causes an amino acid substitution at position 215 of the PhoP protein [83], a residue that is predicted to contact the target DNA [74]. Whereas glycine is encoded at this position (PhoP-G215) in *Y. pseudotuberculosis*, and the ancestral strain of *Y. pestis*, serine (PhoP-S215), is encoded in the strains belonging to branches 1 and 2. A mutational study showed that the wild-type *Y. pestis* KIM strain carrying the *phoP-S215* allele has an increased ability to grow under a low-Mg^2+^ condition, induce the PhoP-regulated gene *ugd*, and resist antimicrobial peptide activity compared to an isogenic strain carrying the ancestral *phoP-G215* allele [74]. The results suggest that this amino acid substitution causes a subtle alteration in PhoP activity and its regulatory network. The evolutionary significance of this change is unknown, as it does not appear to affect the ability of *Y. pestis* to survive inside macrophages or to colonize the flea gut [74]. However, these results raise a possibility that the acquisition of the modern *phoP* allele may have been part of many small genetic changes that contributed to the increased virulence and/or the host adaptability of *Y. pestis*. 

## 7. Summary and Future Perspective

**Figure 2:**
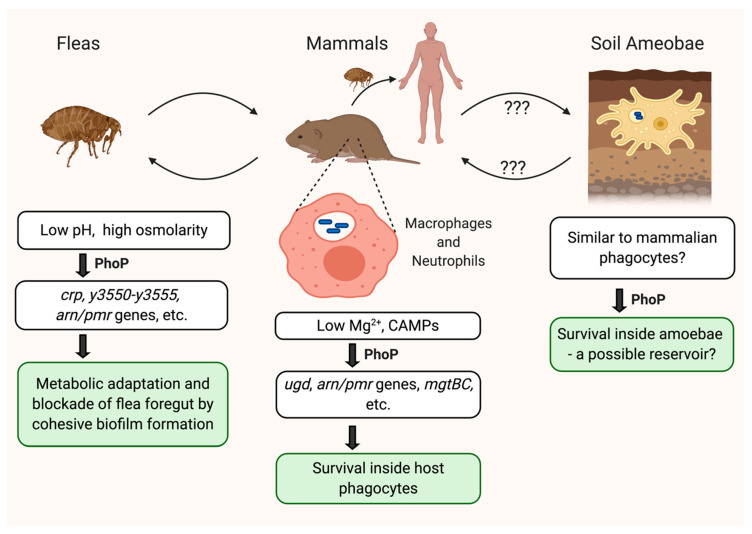
The role of PhoP during different stages of the *Y. pestis* life cycle. PhoP plays a diverse role in the *Y. pestis* life cycle by responding to various stresses the bacteria encounters. Inside the phagosomes of mammalian host macrophages and neutrophils, PhoP is likely activated by the low-Mg^2+^ signal and the presence of CAMPs, and promotes resistance to antimicrobial peptides and other intracellular stresses. Inside the flea gut, PhoP responds to osmotic and acidic stresses, and coordinates changes in bacterial metabolism and formation of a cohesive biofilm necessary for the blockage of flea foregut. The PhoP function is also required for the survival of *Y. pestis* inside the soil-dwelling amoeba *Acanthamoeba castellanii*, suggesting the possibility that the environment inside the phagosomes of the amoeba might be similar to that of mammalian phagocytes. Further studies are needed to define the role of amoeba during the quiescent stage of the *Y. pestis* life cycle. This figure was created with BioRender.com.

PhoP is a versatile transcriptional regulator that is involved in the response to various stresses *Y. pestis* encounters during its life (Figure 2). In mammalian hosts, PhoP plays a crucial role in survival inside host phagocytes by promoting resistance to antimicrobial peptides and other harsh conditions. In fleas, PhoP responds to osmotic and acidic stresses, and coordinates changes in bacterial metabolism and the formation of a cohesive biofilm required for flea blockage. The roles of PhoP during interaction with amoebae need further elucidation, but they may be similar to those observed within mammalian phagocytes. The PhoP regulon in *Y. pestis* appears to have evolved dynamically, incorporating newly acquired genes into its network while keeping the ancestral genes as part of the core regulon, and these evolutionary changes may have facilitated adaptation of the pathogen to different host environments [31]. For example, the low-GC content *y3550–y3555* operon has been predicted to be a direct target of *Y. pestis* PhoP [68], but this operon is not commonly found in bacteria other than *Y. pestis* and *Y. pseudotuberculosis*; among its gene products, only Y3555 is found to have homologs in rare *Salmonella* food-borne isolates [67]. An increased expression of this operon during flea infection suggests that perhaps its incorporation into the *Y. pestis* PhoP regulon may have improved the pathogen’s ability to cope with physiological challenges specific to the flea gut. On the other hand, the core PhoP regulon, including *pmrHFIJKLM*, *ugd*, and *mgtCB*, may encode evolutionarily conserved mechanisms that promote *Y. pestis* intracellular survival in both the soil amoebae and the mammalian phagocytes. Finally, a number of small changes in the PhoP regulon during *Y. pestis* evolution, such as SNPs in *phoP* and the PhoP-target *pagP*, which resulted in a subtle increase in the PhoP transcriptional activity and the alteration of lipid A modification to a less immunostimulatory form, respectively, also may have contributed to the enhanced pathogenicity of the bacteria [24,74]. Further analysis of the PhoP regulon should provide insights into how the dynamic nature of the bacterial regulatory network may contribute to the adaptation of a pathogen to different hosts during evolution.
